# The prognostic significance of *KRAS* and *BRAF* mutation status in Korean colorectal cancer patients

**DOI:** 10.1186/s12885-017-3381-7

**Published:** 2017-06-05

**Authors:** Daeyoun David Won, Jae Im Lee, In Kyu Lee, Seong-Taek Oh, Eun Sun Jung, Sung Hak Lee

**Affiliations:** 1 Department of Surgery, Seoul St. Mary’s Hospital, College of Medicine, The Catholic University of Korea, Seoul, Republic of Korea; 2 Department of Surgery, Uijeongbu St. Mary’s Hospital, College of Medicine, The Catholic University of Korea, Seoul, Republic of Korea; 3 Department of Hospital Pathology, Seoul St. Mary’s Hospital, College of Medicine, The Catholic University of Korea, 222, Banpo-daero, Seocho-gu, Seoul, 06591 Republic of Korea

**Keywords:** *BRAF* mutation, *KRAS* mutation, MSI, Colorectal cancer

## Abstract

**Background:**

*BRAF* and *KRAS* mutations are well-established biomarkers in anti-EGFR therapy. However, the prognostic significance of these mutations is still being examined. We determined the prognostic value of *BRAF* and *KRAS* mutations in Korean colorectal cancer (CRC) patients.

**Methods:**

From July 2010 to September 2013, 1096 patients who underwent surgery for CRC at Seoul St. Mary’s Hospital were included in the analysis. Resected specimens were examined for *BRAF*, *KRAS*, and microsatellite instability (MSI) status. All data were reviewed retrospectively.

**Results:**

Among 1096 patients, 401 (36.7%) had *KRAS* mutations and 44 (4.0%) had *BRAF* mutations. Of 83 patients, 77 (92.8%) had microsatellite stable (MSS) or MSI low (MSI-L) status while 6 (7.2%) patients had MSI high (MSI-H) status. Patients with *BRAF* mutation demonstrated a worse disease-free survival (DFS, HR 1.990, CI 1.080–3.660, *P* = 0.02) and overall survival (OS, HR 3.470, CI 1.900–6.330, *P* < 0.0001). Regarding *KRAS* status, no significant difference was noted in DFS (*P* = 0.0548) or OS (*P* = 0.107). Comparing the MSS/MSI-L and MSI-H groups there were no significant differences in either DFS (*P* = 0.294) or OS (*P* = 0.557).

**Conclusions:**

*BRAF* mutation, rather than *KRAS*, was a significant prognostic factor in Korean CRC patients at both early and advanced stages. The subgroup analysis for MSI did not show significant differences in clinical outcome. BRAF should be included in future larger prospective biomarker studies on CRC.

**Electronic supplementary material:**

The online version of this article (doi:10.1186/s12885-017-3381-7) contains supplementary material, which is available to authorized users.

## Background

Colorectal cancer (CRC) is the second most common cancer in females and the third most common cancer in males worldwide [1]. It is one of the most rapidly growing cancers in Korea with an annual increase (from 1999 to 2009) of 6.2% in men and 6.8% in women [2]. Despite advances in CRC treatment and a decline in the mortality rate over the past few decades, CRC remains the second most common cause of cancer death in females and third common cause of cancer death in males [3].

Considerable advances have been made in the characterization of genetic alterations in CRC in support of genome-wide profiling. The Cancer Genome Atlas Network accomplished the largest comprehensive molecular analysis of CRC to date [4]. Based on somatic mutation rates, colorectal adenocarcinomas were classified as hypermutated or non-hypermutated. The hypermutated group had somatic mutations caused by high microsatellite instability (MSI), usually with *MLH1* silencing or mismatch repair gene mutations. *BRAF* and *ACVR2A* mutations were enriched in hypermutated samples. However, the non-hypermutated group had frequent gene copy number alterations. In addition, *APC*, *TP53*, *KRAS*, and *PIK3CA* mutations were observed. These are characteristic of chromosomal instability [4].

The v-Ki-ras2 Kirsten rat sarcoma viral oncogene homolog (*KRAS*), a member of the Ras subfamily, is a proto-oncogene that encodes a 21 kDa GTPase located on the short arm of chromosome 12 [5]. The RAS protein activates several downstream signaling cascades such as the mitogen-activated protein kinase (MAPK) and PI3K pathways that regulate multiple cellular functions including cell proliferation, differentiation, motility, survival, and intracellular trafficking [6]. KRAS is considered a key downstream component of the epidermal growth factor receptor (EGFR) signaling pathway; therefore, mutations of the gene result in a constitutive activation of the EGFR signaling cascade [5]. *KRAS* mutations are identified in 30–50% of CRCs and are usually point mutations that occur in codons 12 and 13, less often in codon 61, and very infrequently at other sites such as codons 59, 146, 19, or 20 [5, 7]. *KRAS* mutation is a well-established biomarker that predicts resistance to therapy using anti-EGFR monoclonal antibodies in metastatic CRC [8]. However, the prognostic value of *KRAS* mutations in CRC is controversial. Some studies revealed that *KRAS* mutations are associated with poorer prognosis, while others have reported no association [9–12].

The v-Raf murine sarcoma viral oncogene homolog B1 (BRAF) is a serine/threonine kinase that plays a part in cell proliferation, survival, and differentiation; [13]. Activating *BRAF* mutations have been detected in various malignant tumors such as melanoma, papillary thyroid cancer, CRC, ovarian cancer, and hairy cell leukemia [13–15]. In CRC, *BRAF* mutations are reported in 4.7 to 20% of tumors [13, 16]. Usually, *BRAF* and *KRAS* mutations are usually mutually exclusive [17]. The most common *BRAF* mutation, found in over 90% of human cancers, is a glutamic acid for valine substitution at codon 600 in exon 15 (V600E), leading to constitutive activation of the MAPK pathway [18]. The predictive role of *BRAF* mutation in response to anti-EGFR therapy remains uncertain; however, previous studies found that *BRAF* mutations are associated with an adverse clinical outcome, especially in advanced stage CRC [16, 19, 20].

In the present study, we comprehensively investigated *KRAS* and *BRAF* mutation status in Korean CRC patients. In addition, we analyzed the relationship of *KRAS* and *BRAF* mutation with MSI status.

## Methods

### Patients and treatment

We retrospectively reviewed specimens from 1096 consecutive patients who underwent surgical CRC resection at Seoul St. Mary’s Hospital, The Catholic University of Korea, between July 2010 and September 2013. CRC cases with tissue blocks eligible for the KRAS and BRAF mutation testing were included in this study. Two gastrointestinal pathologists reviewed and classified CRC slides according to World Health Organization classification. Clinicopathological parameters were obtained from patient medical records and pathology reports at our institution. Adjuvant chemotherapy was recommended to high-risk (cancer obstruction, perforation, poor differentiation, or lymphovascular/perineural invasion) stage II or stage III CRC patients. According to the *BRAF* and *KRAS* mutational status, patients were offered targeted agents as an adjunct to systemic chemotherapy. However, due to insurance coverage issues, only 3 patients received anti-EGFR and only 12 received anti-vascular endothelial growth factor therapy during the study period. Approval for this study was acquired from the Institutional Review Board of the Catholic University of Korea, College of Medicine (KC16RISI0011).

### DNA isolation and analysis of *KRAS* and *BRAF* mutations

For DNA isolation, 10-μm-thick sections from formalin-fixed paraffin-embedded (FFPE) tissue samples were used for each case. Hematoxylin & eosin sections were used as a reference and the largest tumor area was scraped off with a scalpel under a dissecting microscope. Genomic DNA was extracted using the QIAamp DNA FFPE tissue kit (Qiagen Inc., Valencia, CA) according to the manufacturer’s recommendations. Sanger sequencing was performed using an ABI 3730 automated sequencer (Applied Biosystems, Inc., Foster City, CA), to detect the presence of *KRAS* exon 2 mutations with previously reported primers [21]. Exon 15 of the *BRAF* gene was amplified by polymerase chain reaction (PCR) using the following forward primer (5′-AATGCTTGCTCTGATAGGAAAAT-3′) and reverse primer (5′-TAATCAGTGGAAAAATAGCCTC-3′), resulting in a 209 base pair PCR product. The resultant PCR products were purified using the QIAquick PCR Purification Kit (Qiagen Inc., Valencia, CA) and the appropriate protocol on the QIAcube robotic workstation. Each chromatogram was visually inspected for abnormalities.

### MSI analysis

Five microsatellite markers (BAT-25, BAT-26, D2S123, D5S346, and D17S250) recommended by a National Cancer Institute workshop on MSI determined the microsatellite status [22]. PCR analyses were performed and the shift of PCR products from tumor DNA was compared to normal DNA. Tumors with at least 2 of the 5 microsatellite markers displaying shifted alleles were classified as MSI-H, whereas tumors with only 1 marker exhibiting a novel band were classified as MSI-L. Samples in which all microsatellite markers displayed the same patterns in tumor and normal tissues were classified as MSS; subsequently, MSS and MSI-L tumors were grouped for analyses based on genetic implications [22].

### Statistical analysis

Continuous variables were analyzed by student’s t or Mann-Whitney U test, expressed as the mean ±SD. For categorical variables, χ^2^-test analysis or Fisher’s exact test was used. Survival analysis was performed by the Kaplan-Meier method. Statistical analysis was performed with SPSS software version 18 (SPSS Inc., Chicago, IL) and the R programing language (R Core Team 2015, A language and environment for statistical computing, R Foundation for Statistical Computing, Vienna, Austria, URL http://www.r-project.org/). A *P*-value of <0.05 was considered significant.

## Results

### Patient characteristics according to *KRAS* or *BRAF* mutation status

**Fig. 1:**
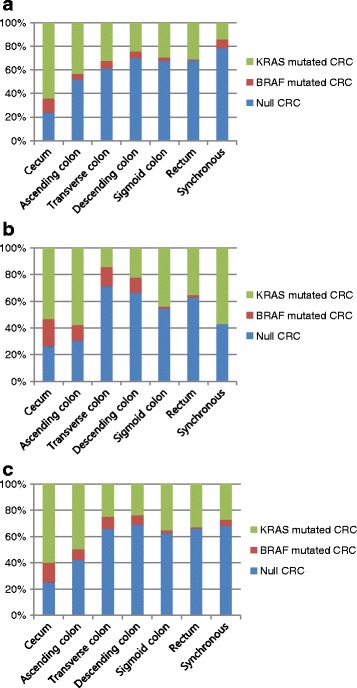
Tumor distribution according to *KRAS* and *BRAF* mutation status. **a** Male patients, **b** Female patients and **c** All patients

The present study included 1092 patients with *KRAS* and 1096 patients with *BRAF* mutation data. Tables 1 and 2 summarize the clinicopathological characteristics of patients. A total of 401 patients (36.7%) had *KRAS* mutations. *KRAS* mutated CRCs were significantly associated with females (45.1% vs 34.6% with wild-type *KRAS*; *P* = 0.001), right sided tumors (32.4% vs 21.0%; *P* < 0.001), higher T stage (T4, 15.3% vs 11.0%; *P* = 0.005), well to moderate differentiation (98.7% vs 94.7%; *P* = 0.002), and mucinous adenocarcinoma (9.2% vs 4.9%; *P* = 0.002). *BRAF* mutations were detected in 44 patients (4.0%). The proportion of *BRAF* mutation was higher in tumors located in the right colon (56.8% vs 23.9% with wild-type *BRAF*; *P* = 0.001), with an advanced tumor stage (T4, 29.5% vs 11.9%; *P* = 0.005), with lymph node metastasis (N2, 38.6% vs 20.5%; *P* = 0.015), and with lymphatic invasion (65.9% vs 44.0%; *P* = 0.007). *BRAF* mutated tumors trended toward poorly differentiated histology (10.0% vs 3.6%, *P* = 0.099) and an infiltrative growth pattern (22.7% vs 15.2%; *P* = 0.065) compared to wild-type *BRAF* tumors, but these were not statistically significant. In addition, gender distribution according to *KRAS* mutation status did not differ significantly, showing a bimodal distribution pattern along the colorectum. Distributions with respect to tumor sites for all three tumor subgroups (*KRAS-*mutated, *BRAF-*mutated and null CRCs), stratified for gender, are shown in Fig. 1a–c.

**Table 1:** Clinicopathologic characteristics according to *KRAS* mutation status

	Patients with *KRAS* status			*p-value*
	Negative	Positive	Total	
	(*N* = 691)	(*N* = 401)	(*N* = 1092)	
Sex				0.001
Male	452 (65.4%)	220 (54.9%)	672 (61.5%)	
Female	239 (34.6%)	181 (45.1%)	420 (38.5%)	
Age				0.771
< 50 year	90 (13.0%)	49 (12.2%)	139 (12.7%)	
≥ 50 year	601 (87.0%)	352 (87.8%)	953 (87.3%)	
Location				<0.001
Rt colon	145 (21.0%)	130 (32.4%)	275 (25.2%)	
Lt colon	309 (44.7%)	158 (39.4%)	467 (42.8%)	
Rectum	221 (32.0%)	107 (26.7%)	328 (30.0%)	
Multiple	16 (2.3%)	6 (1.5%)	22 (2.0%)	
Stage				0.889
Tis	15 (2.2%)	8 (2.0%)	23 (2.1%)	
StageI	129 (18.8%)	75 (18.8%)	204 (18.8%)	
StageII	195 (28.3%)	112 (28.0%)	307 (28.2%)	
StageIII	256 (37.2%)	142 (35.5%)	398 (36.6%)	
StageIV	93 (13.5%)	63 (15.8%)	156 (14.3%)	
T stage				0.005
T1	71 (10.5%)	25 (6.4%)	96 (9.0%)	
T2	100 (14.8%)	77 (19.7%)	177 (16.6%)	
T3	429 (63.6%)	229 (58.6%)	658 (61.8%)	
T4	74 (11.0%)	60 (15.3%)	134 (12.6%)	
N stage				0.897
N0	362 (52.5%)	207 (51.6%)	569 (52.2%)	
N1	184 (26.7%)	106 (26.4%)	290 (26.6%)	
N2	143 (20.8%)	88 (21.9%)	231 (21.2%)	
M stage				0.35
M0	598 (86.5%)	338 (84.3%)	936 (85.7%)	
M1	93 (13.5%)	63 (15.7%)	156 (14.3%)	
Lymphatic invasion				0.163
Absent	392 (56.8%)	209 (52.2%)	601 (55.1%)	
Present	298 (43.2%)	191 (47.8%)	489 (44.9%)	
Venous invasion				0.055
Absent	558 (81.0%)	343 (85.8%)	901 (82.7%)	
Present	131 (19.0%)	57 (14.2%)	188 (17.3%)	
Perineural invasion				0.123
Absent	537 (77.8%)	294 (73.5%)	831 (76.2%)	
Present	153 (22.2%)	106 (26.5%)	259 (23.8%)	
Differentiation				0.002
Well/Moderate	629 (94.7%)	374 (98.7%)	1003 (96.2%)	
Poor	35 (5.3%)	5 (1.3%)	40 (3.8%)	
Histology				0.008
Non-mucinous adenocarcinoma	657 (95.1%)	364 (90.8%)	1021 (93.5%)	
Mucinous adenocarcinoma	34 (4.9%)	37 (9.2%)	71 (6.5%)	
Recur				0.143
Recur	593 (85.8%)	330 (82.3%)	923 (84.5%)	
Non-recur	98 (14.2%)	71 (17.7%)	169 (15.5%)	
Expire				0.219
Expire	629 (91.0%)	355 (88.5%)	984 (90.1%)	
Non- Expire	62 (9.0%)	46 (11.5%)	108 (9.9%)	
Neoadjuvant Tx				0.217
No	605 (87.6%)	364 (90.8%)	969 (88.7%)	
CTx	31 (4.5%)	10 (2.5%)	41 (3.8%)	
RT	2 (0.3%)	0 (0.0%)	2 (0.2%)	
CCRT	53 (7.7%)	27 (6.7%)	80 (7.3%)

**Table 2:** Clinicopathologic characteristics according to *BRAF* mutation status

	Patients with *BRAF* status			*p-value*
	Negative	Positive	Total	
	(*N* = 1052)	(*N* = 44)	(*N* = 1096)	
Sex				*0.149*
Male	652 (62.0%)	22 (50.0%)	674 (61.5%)	
Female	400 (38.0%)	22 (50.0%)	422 (38.5%)	
Age				*0.375*
< 50 year	131 (12.5%)	8 (18.2%)	139 (12.7%)	
≥ 50 year	921 (87.5%)	36 (81.8%)	957 (87.3%)	
Location				*0*
Rt colon	252 (24.0%)	25 (56.8%)	277 (25.3%)	
Lt colon	455 (43.3%)	14 (31.8%)	469 (42.8%)	
Rectum	324 (30.8%)	4 (9.1%)	328 (29.9%)	
Multiple	21 (2.0%)	1 (2.3%)	22 (2.0%)	
Stage				*0.226*
Tis	23 (2.2%)	0 (0.0%)	23 (2.1%)	
StageI	205 (19.6%)	5 (11.4%)	210 (19.2%)	
StageII	323 (30.9%)	12 (27.3%)	335 (30.7%)	
StageIII	496 (47.4%)	27 (61.4%)	523 (47.9%)	
T stage				*0.006*
T1	93 (9.1%)	3 (6.8%)	96 (9.0%)	
T2	173 (16.9%)	4 (9.1%)	177 (16.6%)	
T3	637 (62.1%)	24 (54.5%)	661 (61.8%)	
T4	122 (11.9%)	13 (29.5%)	135 (12.6%)	
N stage				*0.015*
N0	553 (52.7%)	17 (38.6%)	570 (52.1%)	
N1	282 (26.9%)	10 (22.7%)	292 (26.7%)	
N2	215 (20.5%)	17 (38.6%)	232 (21.2%)	
M stage				
M0	3 (75.0%)	0 (0.0%)	3 (75.0%)	
M1	1 (25.0%)	0 (0.0%)	1 (25.0%)	
Lymphatic invasion				*0.007*
Absent	588 (56.0%)	15 (34.1%)	603 (55.1%)	
Present	462 (44.0%)	29 (65.9%)	491 (44.9%)	
Venous invasion				*0.109*
Absent	873 (83.2%)	32 (72.7%)	905 (82.8%)	
Present	176 (16.8%)	12 (27.3%)	188 (17.2%)	
Perineural invasion				*0.451*
Absent	804 (76.6%)	31 (70.5%)	835 (76.3%)	
Present	246 (23.4%)	13 (29.5%)	259 (23.7%)	
Differentiation				*0.081*
Well	96 (9.5%)	2 (5.0%)	98 (9.4%)	
Moderate	875 (86.9%)	34 (85.0%)	909 (86.8%)	
Poor	36 (3.6%)	4 (10.0%)	40 (3.8%)	
Histology				*0.302*
Non-mucinous adenocarcinoma	986 (93.7%)	39 (88.6%)	1025 (93.5%)	
Mucinous adenocarcinoma	66 (6.3%)	5 (11.4%)	71 (6.5%)	
Recur				*0.113*
Recur	894 (85.0%)	33 (75.0%)	927 (84.6%)	
Non-recur	158 (15.0%)	11 (25.0%)	169 (15.4%)	
Expire				*0*
Expire	956 (90.9%)	32 (72.7%)	988 (90.1%)	
Non-Expire	96 (9.1%)	12 (27.3%)	108 (9.9%)	
Neoadjuvant Tx				*0.589*
No	929 (88.3%)	41 (93.2%)	970 (88.5%)	
CTx	40 (3.8%)	2 (4.5%)	42 (3.8%)	
RT	2 (0.2%)	0 (0.0%)	2 (0.2%)	
CCRT	81 (7.7%)	1 (2.3%)	82 (7.5%)

### Mutation frequencies in *KRAS* and *BRAF*

A *KRAS* codon 12 mutation was observed in 296 patients. A *KRAS* codon 13 mutation was observed in 98 patients. Seven other patients had either *KRAS* codon 14 or 30 mutations. The most frequent amino acid change was Gly12Asp, which accounted for 36.9% of KRAS mutations (148/401). The second most frequent mutation was Gly13Asp (24.2%, 97/401), and the third was Gly12Val (21.9%, 88/401). Table 3 lists detailed nucleotide and codon changes**.** Regarding *BRAF* mutations, Val600Glu in exon 15 showed the highest frequency (97.7%, 43/44) (Table 4). In addition, our data revealed 3 *KRAS* and *BRAF* co-mutated cases. Among these 3 cases, 2 had Gly13Asp KRAS mutations, 1 had a Gly12Asp mutation, and all BRAF mutations were Val600Glu. All 3 cases had lymph node metastasis and were included in stage III; however, no recurrences or deaths were observed.

**Table 3:** Frequency of Mutations in *KRAS* exon2

*KRAS* codon 12		
c.34G > A	Gly12Ser	16
c.34G > C	Gly12Arg	2
c.34G > T	Gly12Cys	31
c.35G > A	Gly12Asp	148
c.35G > T	Gly12Asp	1
c.35G > T	Gly12Val	88
c.38G > A	Gly12Asp	5
c.35G > C	Gly12Ala	11
*KRAS* codon 13		
c.35G > A	Gly13Asp	1
c.38G > A	Gly13Asp	97
c.37G > T	Gly13Cys	2
c.36G > T	Gly13Val	2
c.38_39 GC > TT	Gly13Val	1
*KRAS* codon 14		
c.40G > A	Val14lle	1
*KRAS* codon 30		
c.90C > T	Asp30Asp	1

**Table 4:** Frequency of *BRAF* Mutations

*BRAF* codon 600		
c.1799 T > A	Val600Glu	43
c.1796 C > G	Thr599Arg	1

### Impact of *KRAS* and *BRAF* mutations on DFS and OS

**Fig. 2:**
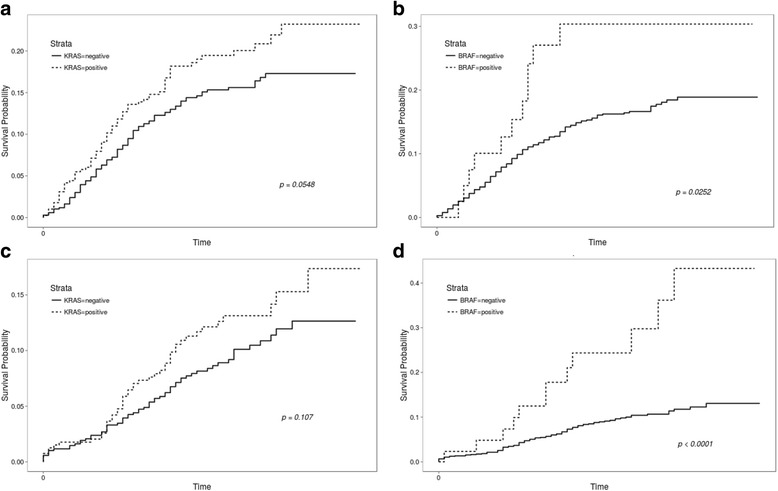
Kaplan-Meier curves for disease-free survival and overall survival according to *KRAS* or *BRAF* mutation status. **a** Disease-free survival (DFS) according to *KRAS* status, **b** DFS according to *BRAF* status, **c** Overall survival (OS) according to *KRAS* status and **d** OS according to *BRAF* status

After a median follow-up of 29 months, the 5-year disease free survival rate of the study population was 81%. There was no significant difference according to *KRAS* mutation status; however, DFS trended toward being shorter in patients with *KRAS* mutations than those with wild-type *KRAS* (*P* = 0.0548). DFS was also significantly worse in patients with *BRAF* mutated cancers compared to wild-type *BRAF* by both univariate (HR 1.98, *P* = 0.0252) and multivariate analyses (HR 2.222) (Fig. 2a and b).

Regarding OS, the 5-year rate was 80%. No significant difference in OS according to *KRAS* mutation status was revealed (*P* = 0.108). OS was significantly shorter for patients with *BRAF* mutations than those with wild-type *BRAF* by univariate analysis (HR 3.46, 95% CI 1.9–6.3, *P* < 0.0001). In the multivariate analysis, *BRAF* mutations also had a negative impact on OS (HR 4.037, 95% CI 2.172–7.506, *P* < 0.0001) (Fig. 2c and d). In addition, we assessed whether the detrimental effect of *KRAS* mutations was different according to mutation subtypes and showed that there were no significant differences in DFS (*P* = 0.931) or OS (*P* = 0.816) (Additional file 1: Fig. S1A and B).

**Fig. 3:**
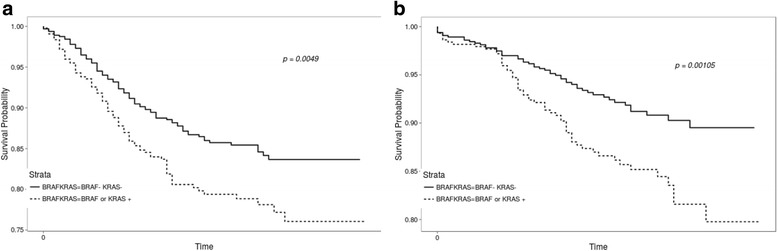
Kaplan-Meier curves for DFS and OS according to *KRAS* mutation status in combination with *BRAF*. **a** DFS according to *KRAS* mutation status in combination with *BRAF* and **b** OS according to *KRAS* mutation status in combination with *BRAF*

Considering *KRAS* and *BRAF* mutations together, DFS and OS were significantly more favorable in patients with wild-type *KRAS* and *BRAF* compared to patients with mutations in both genes (HR 1.540, 95% CI 1.140–2.080, *P* = 0.0049) and OS (HR 1.860, 95% CI 1.280–2.720, *P* = 0.0010) (Fig. 3a and b).

### Subgroup analysis on DFS and OS by stage

**Fig. 4:**
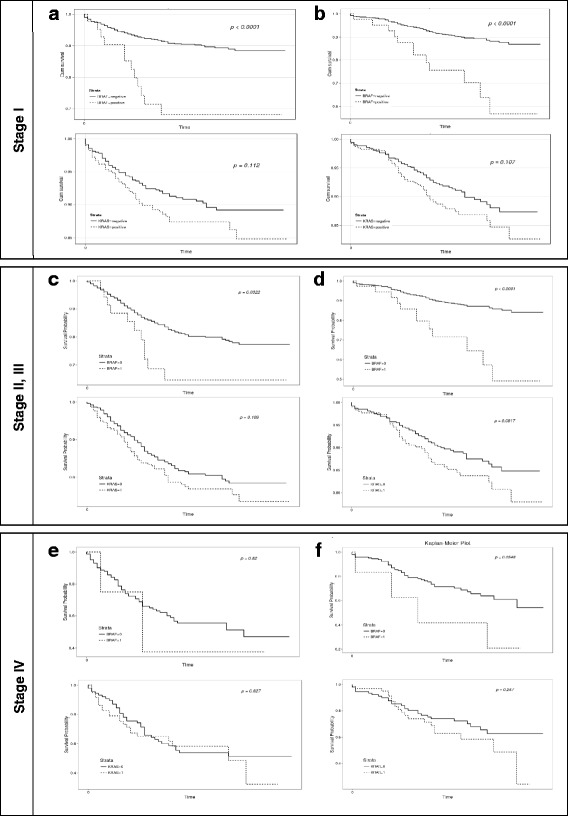
Kaplan-Meier curves for DFS and OS according to *KRAS* or *BRAF* status in CRC patients with different stage. **a** DFS according to *KRAS* or *BRAF* status in CRC patients with stage I, **b** OS according to *KRAS* or *BRAF* status in CRC patients with stage I, **c** DFS according to *KRAS* or *BRAF* status in CRC patients with stage II and III, **d** OS according to *KRAS* or *BRAF* status in CRC patients with stage II and III, **e** DFS according to *KRAS* or *BRAF* status in CRC patients with stage IV and **f** OS according to *KRAS* or *BRAF* status in CRC patients with stage IV

In stage I colorectal cancer, *BRAF* mutations had a negative impact on both DFS (HR 3.936, 95% CI 2.120–7.306, *P* < 0.0001) and OS (HR 4.037, 95% CI 2.172–7.506, *P* < 0.0001). However, *KRAS* mutations did not demonstrate a significant effect on DFS (HR 1.539, 95% CI 1.039–2.279, *P* = 0.112) or OS (HR 1.555, 95% CI 1.048–2.305, *P* = 0.107) (Fig. 4a and b). In stage II and III colorectal cancer, *BRAF* mutations had a negative impact on DFS (HR 1.940, 95% CI 1.050–3.570, *P* = 0.0322) and OS (HR 3.320, 95% CI 1.820–6.070, *P* < 0.0001). However, *KRAS* mutations did not demonstrate a significant effect on DFS (HR 1.250, 95% CI 0.910–1.720, *P* = 0.169) or OS (HR 1.400, 95% CI 0.950–2.070, *P* = 0.0917) (Fig. 4c and d). In stage IV CRC, *BRAF* mutation status did not show a significant effect on DFS (HR 1.180, 95% CI 0.290–4.870, *P* = 0.82) or OS (HR 2.660, 95% CI 0.950–7.450, *P* = 0.0548). *KRAS* mutation status also did not demonstrate a significant effect on DFS (HR 1.140, 95% CI 0.670–1.930, *P* = 0.627) or OS (1.410, 95% CI 0.790–2.520, *P* = 0.247) (Fig. 4e and f).

### Patient characteristics according to MSI status

MSI test data were available in 83 patients. Univariate analysis was performed according to clinicopathologic factors and MSI status. A significant difference was noted in CRC location (*P* = 0.037). MSH-H had a higher frequency in colon cancers of the right side (66.7% vs 23.4%). MSS/MSI-L CRCs were more prevalent on the left (50.6% vs 16.7%). Regarding histological differentiation, a significant difference was noted (*P* = 0.012). MSI-H had higher number of poorly differentiated CRC (1.4% vs 25.0%). Mucinous CRC was observed more frequently in the MSI-H group (6.5% vs 83.3%, *P* < 0.001) (Table 5).

**Table 5:** Clinicopathologic characteristics according to MSI status

	Patients with MSI status			*p-value*
	MSS/MSI-L	MSI-H	total	
	(*N* = 77)	(*N* = 6)	(*N* = 83)	
Sex				*0.482*
Male	44 (57.1%)	2 (33.3%)	46 (55.4%)	
Female	33 (42.9%)	4 (66.7%)	37 (44.6%)	
Age				*0.608*
< 50 year	13 (16.9%)	0 (0.0%)	13 (15.7%)	
≥ 50 year	64 (83.1%)	6 (100.0%)	70 (84.3%)	
Location				*0.037*
Rt colon	18 (23.4%)	4 (66.7%)	22 (26.5%)	
Lt colon	39 (50.6%)	1 (16.7%)	40 (48.2%)	
Rectum	17 (22.1%)	0 (0.0%)	17 (20.5%)	
Multiple	3 (3.9%)	1 (16.7%)	4 (4.8%)	
Stage				*0.642*
StageI	14 (18.2%)	2 (33.3%)	16 (19.3%)	
StageII	27 (35.1%)	2 (33.3%)	29 (34.9%)	
StageIII	36 (46.8%)	2 (33.3%)	38 (45.8%)	
T stage				*0.984*
T1	9 (11.7%)	1 (16.7%)	10 (12.0%)	
T2	13 (16.9%)	1 (16.7%)	14 (16.9%)	
T3	39 (50.6%)	3 (50.0%)	42 (50.6%)	
T4	16 (20.8%)	1 (16.7%)	17 (20.5%)	
N stage				*0.788*
N0	41 (53.2%)	4 (66.7%)	45 (54.2%)	
N1	14 (18.2%)	1 (16.7%)	15 (18.1%)	
N2	22 (28.6%)	1 (16.7%)	23 (27.7%)	
Lymphatic invasion				*0.971*
Absent	46 (59.7%)	3 (50.0%)	49 (59.0%)	
Present	31 (40.3%)	3 (50.0%)	34 (41.0%)	
Venous invasion				*0.378*
Absent	58 (75.3%)	6 (100.0%)	64 (77.1%)	
Present	19 (24.7%)	0 (0.0%)	19 (22.9%)	
Perineural invasion				*0.248*
Absent	53 (68.8%)	6 (100.0%)	59 (71.1%)	
Present	24 (31.2%)	0 (0.0%)	24 (28.9%)	
Differentiation				*0.012*
Well	13 (17.8%)	0 (0.0%)	13 (16.9%)	
Moderate	59 (80.8%)	3 (75.0%)	62 (80.5%)	
Poor	1 (1.4%)	1 (25.0%)	2 (2.6%)	
Histology				<0.001
Non-mucinous adenocarcinoma	72 (93.5%)	1 (16.7%)	73 (88.0%)	
Mucinous adenocarcinoma	5 (6.5%)	5 (83.3%)	10 (12.0%)	
Recur				*0.608*
Recur	64 (83.1%)	6 (100.0%)	70 (84.3%)	
Non-recur	13 (16.9%)	0 (0.0%)	13 (15.7%)	
Expire				*1*
Expire	71 (92.2%)	6 (100.0%)	77 (92.8%)	
Non-Expire	6 (7.8%)	0 (0.0%)	6 (7.2%)	
BRAF status				*0.326*
Wild type	76 (98.7%)	5 (83.3%)	81 (97.6%)	
Mutation	1 (1.3%)	1 (16.7%)	2 (2.4%)	
KRAS status				*0.102*
Wild type	44 (57.1%)	6 (100.0%)	50 (60.2%)	
Mutation	33 (42.9%)	0 (0.0%)	33 (39.8%)	

### Impact of MSI status on DFS and OS

**Fig. 5:**
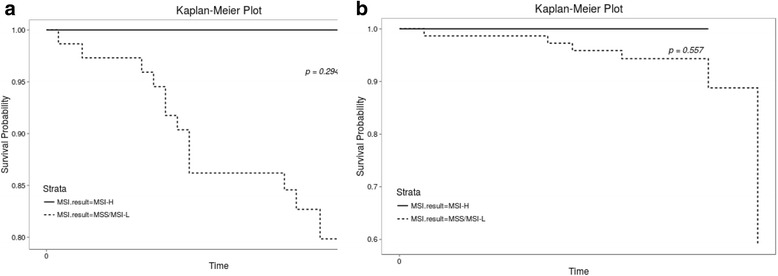
Kaplan-Meier curves for DFS and OS according to MSI status. **a** DFS according to MSI status and **b** OS according to MSI status

We compared DFS and OS between MSS/MSI-L and MSI-H groups to evaluate the value of MSI status as a prognostic marker. MSI status did not show a significant difference in DFS (*P* = 0.294) or OS (*P* = 0.557) (Fig. 5a and b).

## Discussion

In this study, we evaluated *KRAS* and *BRAF* mutational status in 1096 Korean CRC patients using direct sequencing. To the best of our knowledge, our study is one of the first to report the prognostic significance of KRAS and BRAF mutation status in the Korean CRC population. A major strength of this study was the comprehensive subgroup analysis done according to CRC stage and MSI status with a relatively large sample size.

We uncovered an overall *KRAS* mutation rate of 36.7% in colorectal cancers, which was consistent with most previous reports [23–26]. We also found that proximal CRCs had a higher percentage of *KRAS* mutations compared to those at a distal location. This finding is in line with a recent study by Rosty et al. [27]. Furthermore, we found that the frequencies of *KRAS* mutations showed a bimodal distribution pattern along the colorectum. Consistent with previous studies, our data indicated that the frequency of *KRAS* mutated tumors was highest in the cecum (60%) [27, 28]. (Fig. 1a–c) The data emphasized the regional differences between proximal and distal CRCs with respect to clinicopathological and molecular pathogenesis [29]. In addition, we saw a bimodal distribution pattern in both male and female patients, which was different from Rosty et al. who showed that the frequencies of *KRAS* mutated carcinoma were diverse in different colorectal segments between male and female subjects [27]. Like CRCs with *BRAF* mutations, *KRAS*-mutated carcinomas had an increased frequency of the mucinous feature. Several others have also reported this finding [27, 30].

In the current study, we revealed that the G > A transition, followed by G > T transversion were the predominant types of *KRAS* mutations, and the substitution of aspartate for glycine at codon 12 was the most frequent change. Others have also identified the G > A transition and the glycine to aspartate transition on codon 12 as the most frequent type of KRAS activating mutation [31–33]. For codon 13, the 38G > A transition was the most frequent type, which was similar to the findings of other studies [23, 34].

*KRAS* mutations were associated with a higher tumor stage (pT) in this study. However, there were no differences in risk of recurrence, DFS or OS in patients according to their *KRAS* mutation status. These findings are in agreement with those by Rosty et al.; however, the prognostic roles of KRAS mutations are still being debated [27, 34, 35].

The reported frequency of *BRAF* mutations in different populations varies widely. In this study, *BRAF* mutations were found in 4.0% of colorectal cancers, which is slightly lower than previous reports worldwide (Table 6) [36–50]. In general, a lower incidence has been noted in Asian populations such as China, Japan, and Saudi Arabia [37–39]. Interestingly, two studies from Korea showed higher *BRAF* mutation rates of 15.9% and 9.6% [40, 41]. The study cohort by Kim et al. consisted of advanced CRC patients, which might have influenced the higher mutation rate in their study [41]. Ahn et al. used the PNA-clamp real-time PCR method for the detection of *BRAF* mutations, which is known to be superior to direct sequencing in sensitivity and might have caused differences in the mutation rate among study groups [40, 51]. In addition, the enrolled patients of the study by Tsai et al. were under 30 years of age and distinct from other studies [47].

**Table 6:** Studies on *BRAF* mutation status in colorectal cancer patients

Reference (year)	Country	*BRAF* mutation % (n)	*BRAF* mutation type (%)	Methods	Prognostic value	Comments
Pai et al. (2012) [36]	USA	11.0 (20)	V600E (100)	real-time PCR	Significant	Stage I-IV proficient DNA mismatch repair
Kadowaki et al. (2015) [37]	Japan	4.9 (40)	V600E (80)	PCR combined with restriction enzyme digestion	Significant	Stage I-III independent of MSI status
Chen et al. (2014) [38]	China	4.2 (9)	V600E (88.9)	direct sequencing	Significant	Stage I-IV
Siraj et al. (2014) [39]	Saudi Arabia	2.5 (19)	V600E (89.5)	direct sequencing	No prognostic significance	Stage I-IV
Ahn et al. (2014) [40]	Korea	15.9 (26)	V600E (100)	PNA clamp real-time PCR	Significant	Stage I-IV
Kim et al. (2014) [41]	Korea	9.6 (13)	N/A	direct sequencing	Significant	Stage III-IV
Yaeger et al. (2014) [42]	USA	5 (92)	V600E (96.7)	mass spectrometry-based assay	Significant	Metastatic colorectal cancers
Eklof et al. (2013) [43]	Sweden	17.9 (35) 13.2 (54)	V600E (100)	allelic discrimination assay	Significant No prognostic significance	Stage I-IV two different cohorts
Renaud et al. (2015) [44]	France	10.6 (19)	V600E (100)	direct sequencing	Significant	Metachronous lung metastasis
de Cuba et al. (2015) [45]	Netherlands	51.0 (73)	V600E (100)	high resolution melting and sequencing	Significant	Stage II and III microsatellite instable colon cancers
Foltran et al. (2015) [46]	Italy	5.2 (10)	V600E (100)	pyrosequencing	Significant	Metastatic colorectal cancers
Tsai et al. (2015) [47]	Taiwan	18.6 (11)	V600E (100)	direct sequencing	Significant	Stage I-IV early-onset colorectal cancers
Saridaki et al. (2013) [48]	Greece	8.2 (41)	V600E (100)	real-time PCR	Significant	Metastatic colorectal cancers
Kalady et al. (2012) [49]	USA	11.7 (56)	V600E (98.2)	direct sequencing	Significant	Stage I-IV
Farina-Sarasqueta et al. (2010) [50]	Netherlands	19.9 (59)	V600E (100)	real-time PCR	Significant	Stage II and III independently of disease stage and therapy.
Present case	Korea	4.0 (44)	V600E (97.7)	direct sequencing	Significant	Stage I-IV Significant prognostic implications through all stages

In this study cohort, we revealed that *BRAF* mutation was significantly associated with poorer DFS and OS in colorectal cancers. In addition, *BRAF* mutational status was an independent prognostic factor for DFS and OS in multivariate analysis, which is consistent with previous studies (Table 5). Moreover, we compared different tumor stages and found that *BRAF* mutations were also associated with poorer DFS and OS in both stage I and stage II/III subgroups. However, there was no significant association between *BRAF* mutation and survival in the stage IV subgroup. Yaeger et al. recently showed that *BRAF* mutation confers a poor prognosis in metastatic CRC patients [42]. This discrepancy may come from the relatively small study population in this metastatic setting, ethnic distinctions and subsequent differences in *BRAF* mutation rates. Further studies in a larger population data are needed to confirm this result. Nevertheless, our findings highlight that the clinical meaning of *BRAF* mutation is similar to Korean CRC patients, even if the mutation frequency is lower than in western patients. Importantly, we revealed that *BRAF* mutation status is important in predicting the prognosis of early CRCs, which is one of the novel findings of our study. Our findings support a role for *BRAF* mutation in the natural history of CRC because only rare cases in our study cohort received targeted therapy other than the standard chemotherapy regimen after resection.

We found that only 0.3% (*n* = 3) of *KRAS* mutated CRC cases harbored *BRAF* mutations. Of these, two cases showed *KRAS* mutations at codon 13 (38G > A) with the remaining mutation at codon 12 (35G > A), and all three cases had the BRAF V600E mutation. The concomitant occurrence of *KRAS* and *BRAF* mutations is very rare in CRCs (< 1%), which imply tha they may play a role in different tumor subtypes [11, 52].

We analyzed the MSI status in 83 CRC patients and revealed a frequency of 7.2% for MSI-H, which appears somewhat lower than reports from western countries [53]. In line with our findings, a recent multicenter study by Oh et al. showed low frequencies of MSI-H in Korean CRC patients [53]. This result suggested ethnic differences in the molecular characteristics of colorectal tumorigenesis including MSI status. MSI is known to be associated with better clinical outcome in early stage CRCs than MSS cancers [54, 55]. In the present study, MSI status did not have significant prognostic value on DFS and OS; however, a tendency toward worse survival was observed in MSS and MSI-L cases.

*BRAF* activating mutations correlated with poor survival in MSS CRC. *BRAF* mutations occur in about 40% of MSI CRCs; however, it was unclear if it had a prognostic impact in this setting [45]. A recent study revealed that both *BRAF* and *KRAS* mutations are associated with poorer survival in MSI CRC patients compared to those with wild-type *BRAF* and *KRAS* genes [45]. However, we could not draw any meaningful conclusion about the *BRAF* and/or *KRAS* status in MSI CRC cohorts because the mutated cases in this study were rare.

A limitation of this study is the insufficiency of data on the efficacy of an EGFR-blocking antibody according to *KRAS* and *BRAF* mutation status due to only rare cases being treated by EGFR targeted therapy at our institution during the study period. In addition, the sample size was too small to evaluate the significance of the MSI status with infrequent *KRAS* and *BRAF* mutation subtypes. Subsequent translational studies from different cohorts are needed to confirm our data. Nevertheless, a strong point of this study is the relative large study cohort which reduce selection bias. We revealed *BRAF* mutation as an independent prognostic marker for CRCs throughout all stages.

## Conclusion

In conclusion, our study demonstrated that *BRAF* mutation, occurring at a low frequency, was a significant prognostic factor in Korean CRC patients. Our data suggests that molecular features that include *KRAS* and *BRAF* mutations as well as MSI status in CRC patients are important in future clinical trials. Further large translational studies are required to validate the significance of both *BRAF* and/or *KRAS* mutation status in MSI CRCs.

## Additional files

Additional file 1: Fig. S1. Kaplan-Meier curves for DFS and OS between *KRAS* mutation at codon 12 and 13. A. DFS between *KRAS* mutation at codon 12 and 13 and B. OS between *KRAS* mutation at codon 12 and 13. (PPTX 266 kb)

## Abbreviations

BRAF: v-Raf murine sarcoma viral oncogene homolog B1
CI: Confidence interval
CRC: Colorectal cancer
DFS: Disease free survival
EGFR: Epidermal growth factor receptor
FFPE: Formalin-fixed paraffin-embedded
KRAS: v-Ki-ras2 Kirsten rat sarcoma viral oncogene homolog
MAPK: Mitogen-activated protein kinase
MSI: Microsatellite instability
OS: Overall survival
