# Supplementary figures and images for: The prognostic significance of KRAS and BRAF mutation status in Korean colorectal cancer patients
Source: BMC Cancer. 2017 Jun 5;17:403. doi: 10.1186/s12885-017-3381-7 (PMC5460473; doi:10.1186/s12885-017-3381-7)

## Slide 1
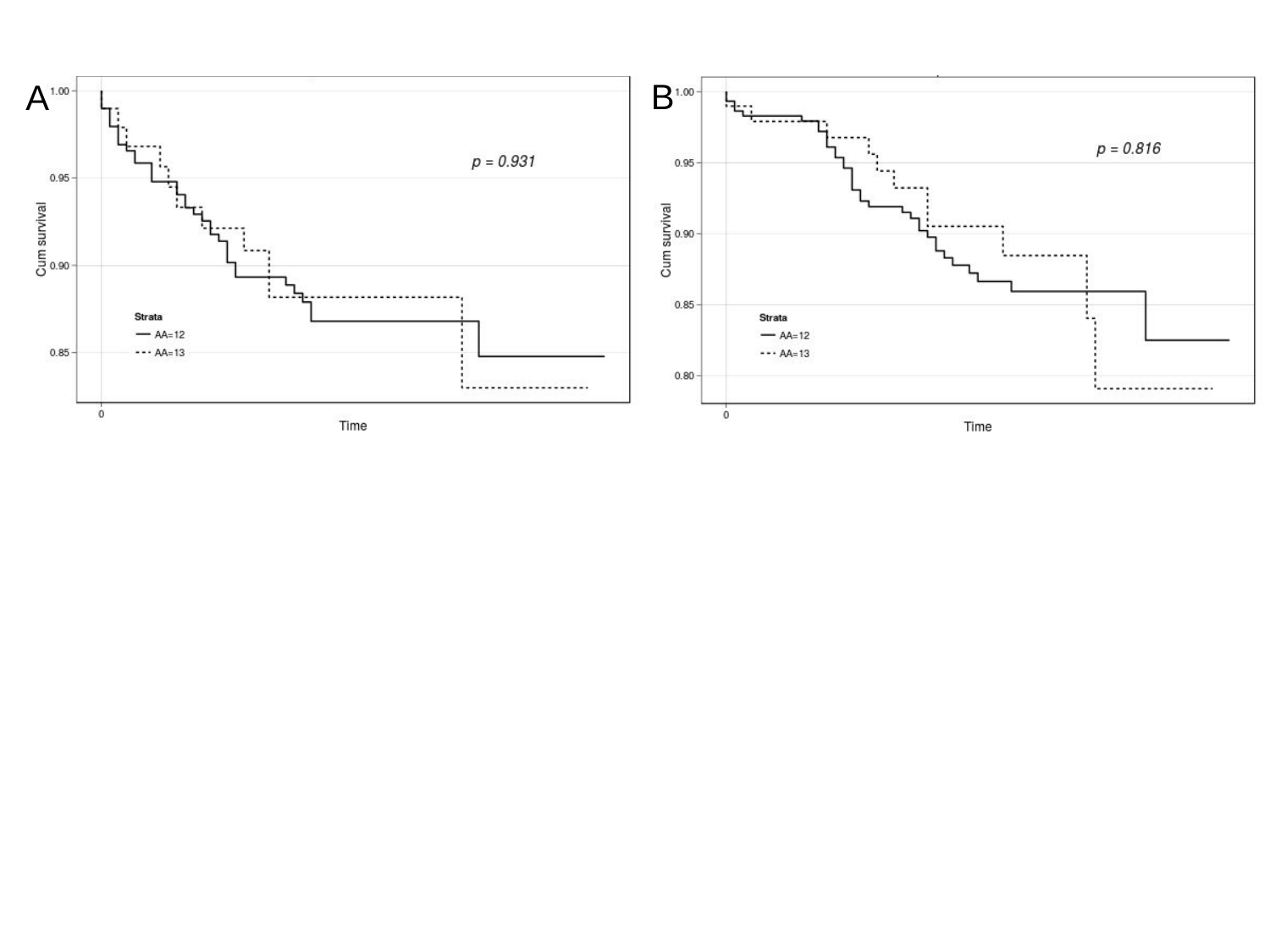

B
A

Supplement: Supplementary file 1 — Kaplan-Meier curves for DFS and OS between KRAS mutation at codon 12 and 13. A. DFS between KRAS mutation at codon 12 and 13 and B. OS between KRAS mutation at codon 12 and 13. (PPTX 266 kb) [file 12885_2017_3381_MOESM1_ESM.pptx]
